# Protease Expression Levels in Prostate Cancer Tissue Can Explain Prostate Cancer-Associated Seminal Biomarkers—An Explorative Concept Study

**DOI:** 10.3390/ijms18050976

**Published:** 2017-05-04

**Authors:** Jochen Neuhaus, Eric Schiffer, Ferdinando Mannello, Lars-Christian Horn, Roman Ganzer, Jens-Uwe Stolzenburg

**Affiliations:** 1 Department of Urology, Research Laboratory, University of Leipzig, Liebigstraße 19, 04103 Leipzig, Germany; 2 Numares AG, Regensburg, Am BioPark 9, 93053 Regensburg, Germany; eric.schiffer@numares.com; 3 Department of Biomolecular Sciences, University “Carlo Bo”, Via O. Ubaldini 7, 61029 Urbino (PU), Italy; ferdinando.mannello@uniurb.it; 4 Institute of Pathology, University Hospital Leipzig, Liebigstraße 24, 04103 Leipzig, Germany; Lars-Christian.Horn@uniklinik-leipzig.de; 5 Department of Urology, University Hospital Leipzig, Liebigstraße 20, 04103 Leipzig, Germany; Roman.Ganzer@uniklinik-leipzig.de (R.G.); Jens-Uwe.Stolzenburg@uniklinik-leipzig.de (J.-U.S.)

**Keywords:** seminal plasma biomarkers, matrix metalloproteinase (MMP), tissue inhibitor of MMP (TIMP), qPCR, confocal laser scanning microscopy, Western blotting

## Abstract

Previously, we described prostate cancer (PCa) detection (83% sensitivity; 67% specificity) in seminal plasma by CE-MS/MS. Moreover, advanced disease was distinguished from organ-confined tumors with 80% sensitivity and 82% specificity. The discovered biomarkers were naturally occurring fragments of larger seminal proteins, predominantly semenogelin 1 and 2, representing endpoints of the ejaculate liquefaction. Here we identified proteases putatively involved in PCa specific protein cleavage, and examined gene expression and tissue protein levels, jointly with cell localization in normal prostate (nP), benign prostate hyperplasia (BPH), seminal vesicles and PCa using qPCR, Western blotting and confocal laser scanning microscopy. We found differential gene expression of chymase (CMA1), matrix metalloproteinases (MMP3, MMP7), and upregulation of MMP14 and tissue inhibitors (TIMP1 and TIMP2) in BPH. In contrast tissue protein levels of MMP14 were downregulated in PCa. MMP3/TIMP1 and MMP7/TIMP1 ratios were decreased in BPH. In seminal vesicles, we found low-level expression of most proteases and, interestingly, we also detected TIMP1 and low levels of TIMP2. We conclude that MMP3 and MMP7 activity is different in PCa compared to BPH due to fine regulation by their inhibitor TIMP1. Our findings support the concept of seminal plasma biomarkers as non-invasive tool for PCa detection and risk stratification.

## 1. Introduction

Prostate cancer (PCa) is the main gender-specific malignancy in men and prostate specific antigen (PSA) testing is the gold standard in PCa detection [1]. However, comprehensive PSA screening resulted in significant overdiagnosis and overtreatment [2] and in consequence PSA screening is recommended only for men aged 55 to 69 years by the AUA [3]. New PCa biomarkers are urgently needed to identify patients who may be candidates for curative intervention and guide clinical decisions [4].

Recently, seminal fluid has been acknowledged as a promising source of PCa related biomarkers as it directly reflects the pathological processes within the prostate [5]. The high biological variability of prostate cancer [6] necessitates a distinct and clearly defined set of biomarkers, rather than a single or a combination of few biomarkers for efficient description of the disease on a molecular level [7]. In a previous study, we defined distinct panels of small peptide biomarkers in seminal plasma by capillary electrophoresis mass spectrometry (CE-MS), which could be used for PCa detection and stratification [8]. The procedure distinguished PCa from benign prostate hyperplasia (BPH), chronic prostatitis (CP) and normal prostate (nP) with 83% sensitivity and 67% specificity (AUC 75%, *p* < 0.0001) in a small clinical validation cohort of 125 patients. A further set of biomarkers correctly identified advanced (≥pT3a) and organ-confined (<pT3a) tumors (AUC 83%, *p* = 0.0055) with 80% sensitivity and 82% specificity [8].

The discovered biomarkers were fragments of larger parental seminal proteins, such as *N*-acetyl lactosaminide β-1,3-*N*-acetyl glucosaminyl transferase, prostatic acid phosphatase, stabilin-2 and most dominantly semenogelin-1 and -2. Although the parental proteins were also detected earlier [9,10,11], the use of these naturally occurring fragments reflecting natural proteolytic liquefaction is a new concept. 

There are mutual activation and inhibition mechanisms within the liquefaction cascade, which could lead to different downstream proteolytic cleavage patterns [12]. The investigation of these disease-associated proteinase networks [13] represents a challenging task to close the gap between the classical protein expression based biomarker concept and the hypothesis-free proteomic profiling concepts. 

We suggest that seminal polypeptide panels may be the smoking gun that links the complex proteolytic alterations of seminal proteins to PCa providing novel insights into PCa biology with special emphasis on disease-associated proteolytic activities. The aim of this study was to identify potential proteinases and inhibitors involved in the formation of the specific seminal biomarkers. 

## 2. Results

### 2.1. Sequence Analysis and Putative Proteolytic Cleavage Sites

Of the 141 seminal peptides discovered in our previous study, representing a total of 47 different parental proteins, almost 60% were fragments of semenogelin-1 or -2 (SEMG1 and SEMG2). Therefore, we focused our data base searches on SEMG1 and SEMG2 cleavage patterns. Using MEROPS database (Available online: http://merops.sanger.ac.uk/cgi-bin/specsearch.pl) we could assign SEMG1 (316–344) to kallikrein-3 (KLK3, PSA) cleavage at site 315 (peptide-SSIY//SQTE-peptide). Limiting searches from octa- to hexamere recognition (peptide-IY//SQ-peptide) resulted in chymase (CMA1) and cathepsin G (CTSG) as potential alternative proteinases (Table 1). In contrast, KLK3 activity may not account for biomarker SEMG2 (194–215) cleaved at position 193 (peptide-SQSS//YVLQ-peptide. Searching cleavage site 193 (peptide-SS//YV-peptide) revealed matrix metallopeptidase-3 (MMP3), -7 (MMP7), -13 (MMP13), -14 (MMP14) or -20 (MMP20) as potentially involved proteases (Table 1). KLK3 activity may also promote for cleavage between Q and T at position 197 (peptide-QVLQ//TEEL-peptide), resulting in biomarkers SEMG1 (198–215) and SEMG2 (198–215) (Table 1). Although KLK3 Q//T-cleavage is reported for SMG1 and SMG2 at position 235, position 197 remains inconclusive (see MEROPS database). KLK cleavage of SEMG1 and SEMG2 at position 194 (peptide-QSSY//VLQT-peptide) between Y and V, results in the observed non-marker fragments starting from position 195 (Table 1).

### 2.2. Gene Expression of Proteases in Human Prostate Samples

**Figure 1:**
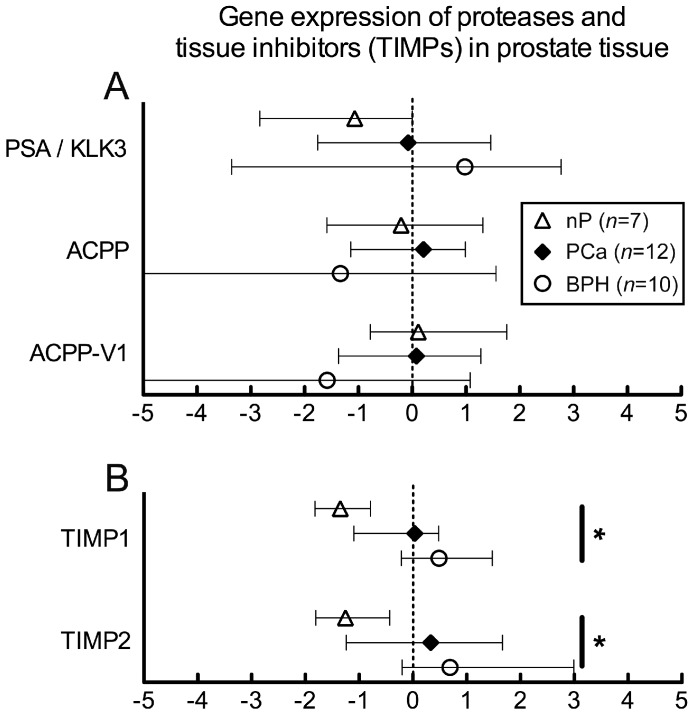

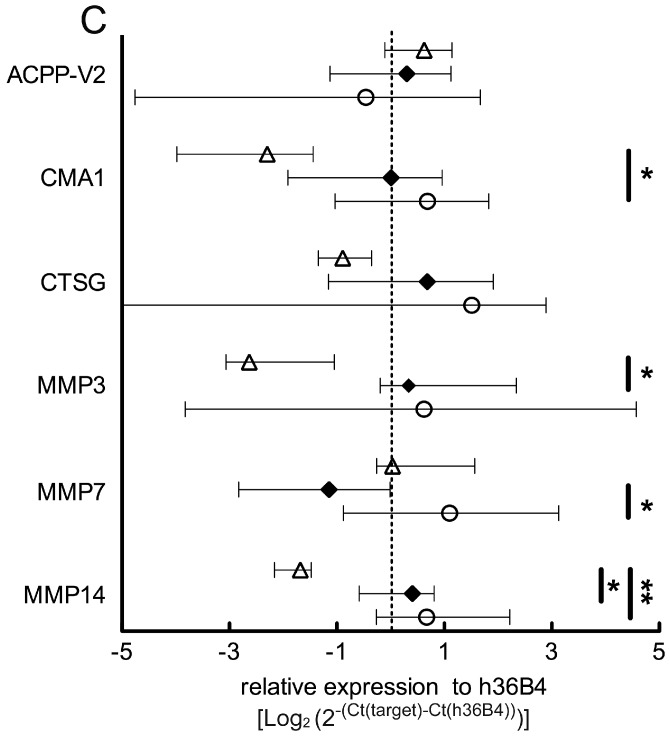
Gene expression analysis. (**A**–**C**) Expression levels compared to h36B4, global normalization; (**A**) High abundance genes (ratio > 1.0); KLK3 (PSA): kallikrein related peptidase 3; ACPP: total prostatic acid phosphatase; ACPP-V1: splice variant 1 (short secreted isoform of ACPP). The observed differences in mRNA levels between PCa (*n* = 12), benign prostatic hyperplasia (BPH) (*n* = 10) and normal Prostate (nP; *n* = 7) were not significant. However, range of KLK3 expression was much higher in BPH and PCa; (**B**) TIMP1 and TIMP2 expression was significantly different between BPH and nP samples; (**C**) Low abundance genes (ratio < 1.0); ACPP-V2: splice variant 2 (long intracellular isoform); CMA1: chymase 1, CTSG: cathepsin G, and MMP: matrix metalloproteinase. Differential expression levels were detected for CMA1, MMP3, MMP7 and MMP14. Symbols represent medians, whiskers indicate interquartile range. Global normalization; interquartile range: 25%–median–75%; significance: *Kruskal–Wallis* test and *Dunn’s* multiple comparison test; * *p* < 0.05, ** *p* < 0.01).

Gene expression of KLK3, prostatic acid phosphatase (ACPP) and ACPP-V1 (isoform 1) was high, while matrix metalloproteinases MMP3, MMP7, MMP14 showed low abundance expression by qPCR (Figure 1). KLK3 showed a tendency of increased levels in BPH (Figure 1A). Significant higher transcript levels in BPH were found for MMP7, MMP14 and CMA1 compared to PCa (Figure 1C). Some of the low abundance genes (CTSG, MMP3) also showed a tendency for higher expression in BPH (Figure 1B). MMP13 and MMP20 were at the detection limits (data not shown).

### 2.3. Protein Levels of Proteinases in Human Prostate and Seminal Vesicles

**Figure 2:**
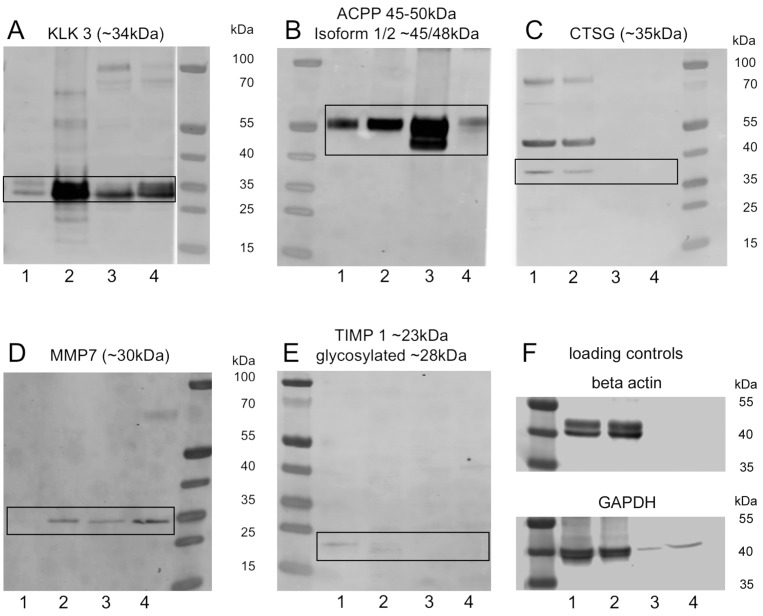
Western blot analysis of prostate and seminal plasma. KLK3 and ACPP were detected in high abundance in prostate tissue (**lanes 1**, **2**) and seminal plasma (**lanes 3**, **4**) at the predicted molecular weight of 34 and 50 kDa, respectively (**A**,**B**); occasionally multiple banding indicated isoforms or post-translational modification (glycosylation); CTSG was detected exclusively in prostate tissue showing three to four distinct bands at ~35, ~45; ~60 and ~80 kDa (**C**); MMP7 could be detected in prostate tissue and seminal plasma at ~30 kDa (**D**) and we found very low levels of TIMP1 (**E**). MMP14, MMP3, TIMP2 and CMA1 were negative in all examined samples (data not shown; *n* = 4 prostate; *n* = 6 seminal plasma); (**F**) loading controls; note negative staining for β actin in seminal.

**Figure 3:**
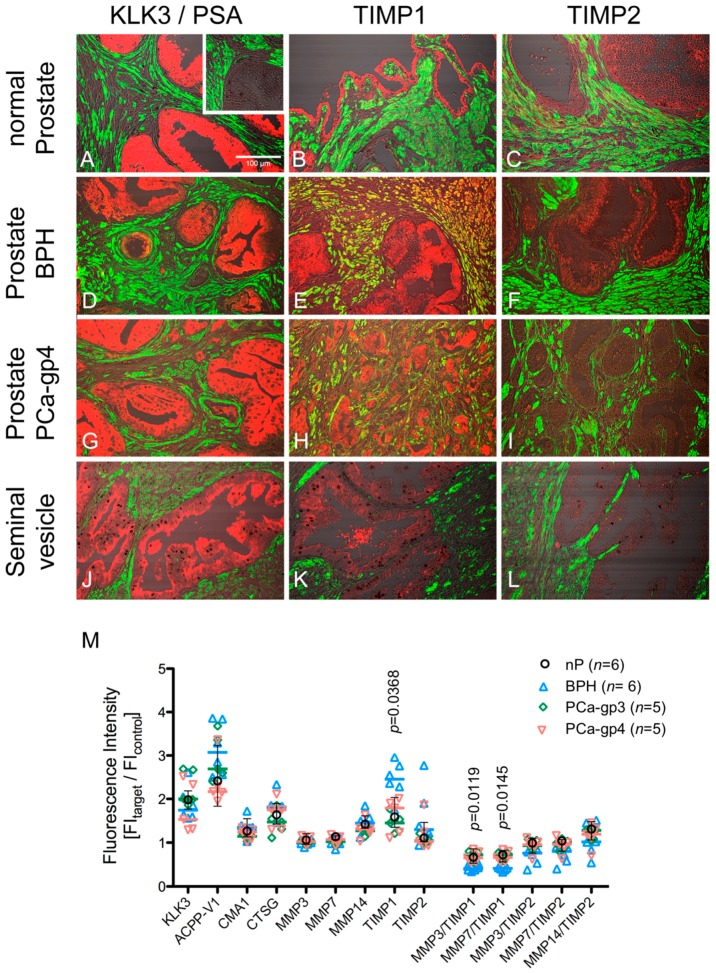
Protein expression analysis using confocal immunofluorescence imaging. Immunohistochemical tissue expression of selected proteases and tissue inhibitors of metalloproteinases. (**A**–**L**) Confocal images demonstrating tissue distribution of target proteins (red); alpha-smooth muscle cell actin (aSMCA; green). Phase contrast images are merged for tissue structure. Immunofluorescence (IF) of KLK3, TIMP1 and TIMP2 in normal prostate (**A**–**C**), BPH (**D**–**F**); PCa Gleason pattern 4 (PCa-gp4; **G**–**I**) and seminal vesicle (**J**–**L**). Note the marked upregulation of TIMP1 in BPH especially in SMC (**E**; orange colored cells) and the downregulation of TIMP1-IF in the smooth muscle cells of PCa-gp4 (**H**); TIMP2-IF is almost completely lost in PCa-gp4 tissue (**I**); KLK3-IF in seminal vesicle was low, TIMP1-IF moderate and TIMP2-IF was very low (**J**–**I**); Scale bar indicating 100 µm in (**A**) applies to all micrographs; staining control (inset in **A**); (**M**) Scatter plot of fluorescence intensities; lines indicate medians. Normal prostate (black symbols) are depicted as median with interquartile range. *p*-Values are based on nonparametric *Kruskal–Wallis* test.

**Figure 4:**
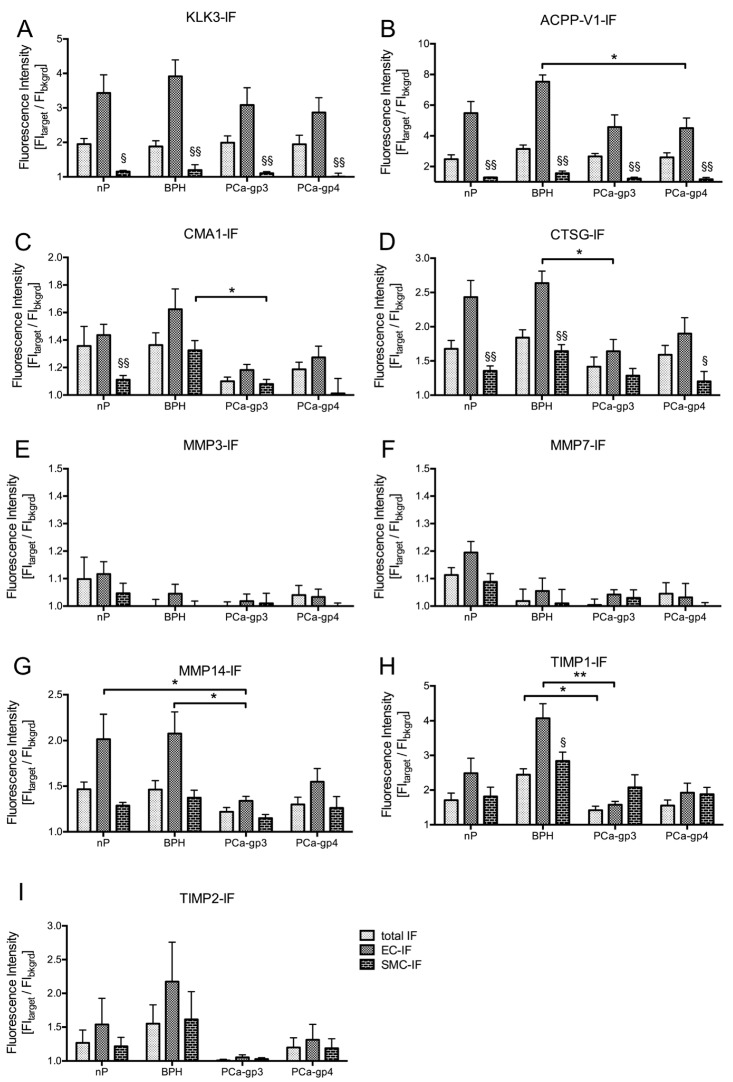
Cellular distribution in prostate tissue. Data (mean (SEM)) are presented as fluorescence intensity [FI_target_/FI_control_]; background fluorescence (bkgrd) = 1. Total fluorescence included complete tissue area omitting the lumen. Smooth muscle cells (SMC) were identified by anti-smooth muscle cell actin staining (green fluorescence, cf. Figure 2); epithelial cells (EC) area was delineated from DIC images. Note the differences in Y-axis scaling. Differences in fluorescence intensities were not significant for KLK3-IF (**A**), MMP3-IF (**E**), MMP7-IF (**F**) and TIMP2-IF (**I**). Note that the total-IF and SMC-IF is unaltered for ACPP-V1 (**B**), CMA1 (**C**) and CTSG (**D**) whereas epithelial (EC)-IF is significantly lower in PCa compared to BPH, indicating contribution of interstitial cells to total-IF. A comparable trend not reaching significance level (*p* < 0.05) is seen for MMP14 (**G**). In contrast, the significant reduction of TIMP1 total-IF is reflected by significant reduction of EC-IF (**H**). *Kruskal–Wallis* test with post-hoc Dunn’s Multiple Comparison test as indicated by bars (* *p* < 0.05; ** *p* < 0.01); § indicates significant difference between EC and SMC; ^§^ *p* < 0.05; ^§§^ *p* < 0.01.

Highest tissue levels were seen for KLK3 and ACPP-V1 by Western blotting (Figure 2A,B; lanes 1 and 2) and epithelial cells showed highest immunofluorescence in CLSM (Figure 3A,M and Figure 4A,B). Differences in total IF among groups were not significant (Figure 3M), except for TIMP1-IF (*p* = 0.0368) as were the calculated ratios: MMP3/TIMP1 (*p* = 0.0119) and MMP7/TIMP1 (*p* = 0.0145; Figure 3M). TIMP1-IF was especially high in epithelium and in interstitium of the prostate, comprising smooth muscle cells (orange labelling in Figure 3E) and interstitial cells (red labelling) in BPH, while being markedly diminished in epithelium and interstitial cells in PCa (Figure 3H). ACPP-V1 (short, secreted isoform of ACPP) showed significant lower immunofluorescence in grade 4 PCa (Figure 4B). Differences between MMP3-IF, MMP7-IF and TIMP2 were not significant (Figure 4C,D,I), whereas trends of lower TIMP2-IF compared to nP were evident in PCa-gp3 (Figure 4I). Total MMP14-IF was lower in PCa-gp3 (*p* < 0.05) and in PCa-gp4 (trend, not significant), and TIMP1-IF was significantly higher in epithelial cells of BPH compared to PCa-gp3 (Figure 4H).

CLSM clearly revealed the different contributions of the epithelial cells and smooth muscle cells to the observed alterations in total IF. Interestingly, KLK3-IF (=PSA) was not different between groups (Figure 4A). Also, MMP3-IF and MMP7-IF showed no differences (Figure 4E,F) whereas MMP14-IF was lower in PCa-gp3 than in nP (Figure 4G); and significant differences were detected for TIMP1-IF (Figure 4H). TIMP2-IF was low in PCa-gp3 but did not reach statistical significance (Figure 4I).

**Figure 5:**
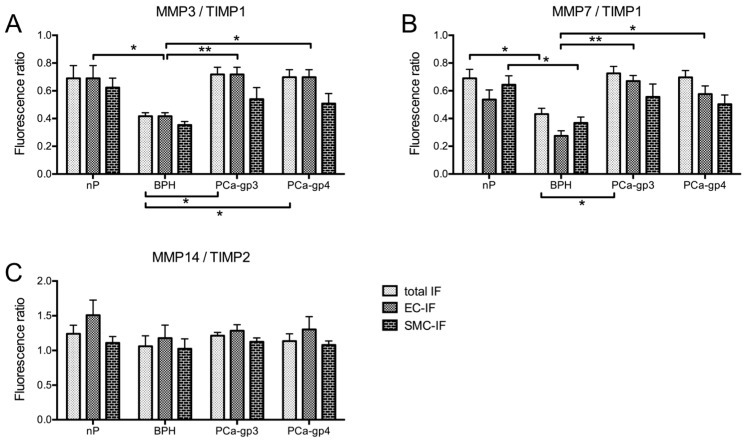
Cellular distribution of MMP/TIMP ratios. Ratios (mean (SEM)) were calculated from means of FI_target_/FI_bkgrd_ of the respective MMP-TIMP pairs and presented as FI-ratio. Note that MMP14/TIMP2 ratios are >1.0 indicating excess of MMP14 (**C**), while MMP3/TIMP1 and MMP7/TIMP1 ratios are <1.0 indicating excess of TIMP1 (**A**,**B**). *Kruskal–Wallis* test with post-hoc *Dunn’s* Multiple Comparison test as indicated by bars (* *p* < 0.05; ** *p* < 0.01); no significant differences were observed between EC and SMC.

We calculated the specific MMP/TIMP ratios as a surrogate of MMP activity. We found that MMPs and TIMP2 were almost balanced in case of MMP3 and MMP7 (values around 1.0; data not shown), while TIMP1 was present in excess indicated by ratios < 1.0 (Figure 5A,B). In contrast, expression of MMP14 well exceeded TIMP2 in nP and PCa as indicated by ratios > 1.0 (Figure 5C), while expression in BPH was balanced.

**Figure 6:**
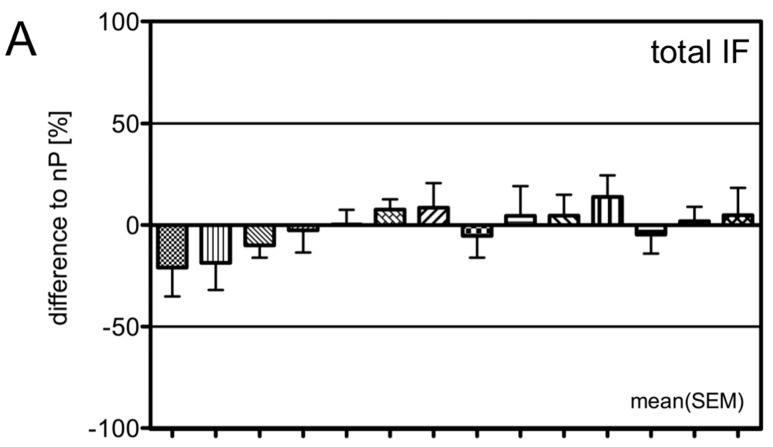

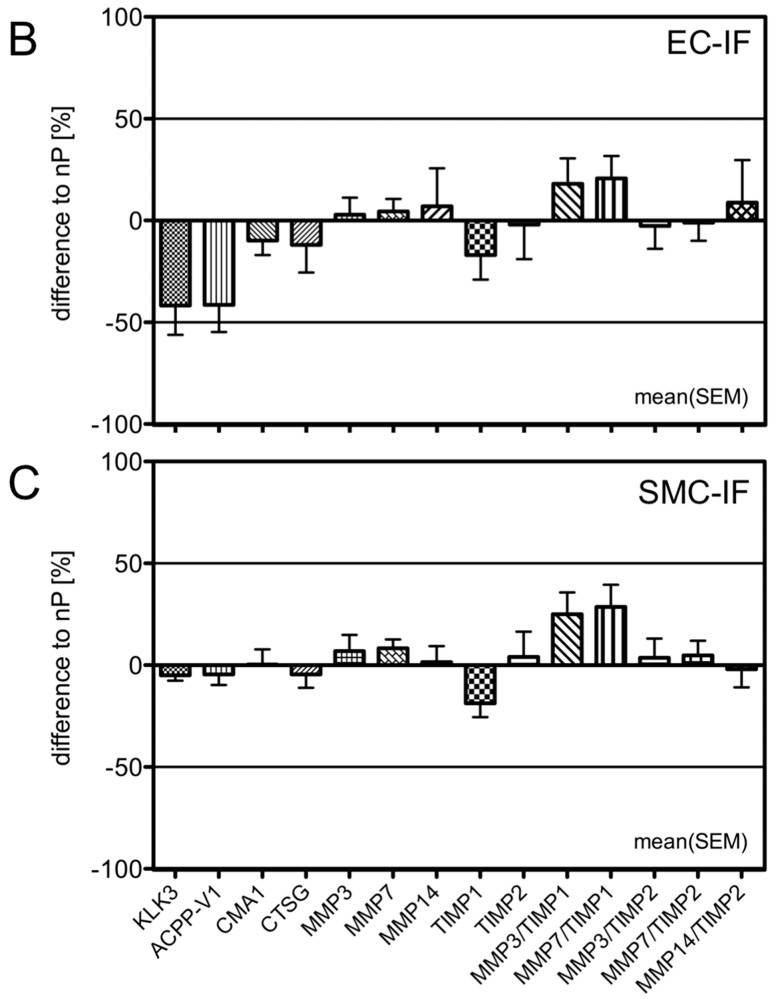
CLSM analysis in seminal vesicles (SV). (**A**–**C**) Immunofluorescence in SV (*n* = 6) was normalized to the IF in nP (*n* = 6). Parameter-free *Mann–Whitney* test detected no significant differences between expression levels in nP and SV, but considerably lower IF was found for KLK3 and ACPP-V1, especially in epithelial cells (EC, **B**).

To address the contribution of seminal vesicles in protease and inhibitor production, we also investigated seminal vesicles (SV) by CLSM and compared the expression to nP. We found no significant differences (*Mann–Whitney* test) but considerably lower IF for KLK3 and ACPP-V1 especially in epithelial cells (EC IF, Figure 6).

## 3. Discussion

Based on our previous study, which defined small protein fragments within the seminal plasma as biomarker fingerprint of prostate carcinoma [8], we were interested in the molecular background of the production of these small peptides (≤20 kDa). We therefore explored the cellular expression of certain proteases and their specific inhibitors within the prostate and seminal vesicle tissues. Most of the small peptides that make up the biomarker pattern were derived from SEMG1 and SEMG2. Cleavage site analysis revealed, that the observed SEMG1 and SEMG2 derived biomarkers cannot be explained by KLK3 cleavage alone, implicating the presence of additional cleavage events. From the available sequence data and proteolytic cleavage site searches we hypothesized proteolysis schemes for SEMG1 and SEMG2 based on the involvement of KLK3, CMA1, CTSG, MMP3, MMP7, MMP13, MMP14 or MMP20 as well as the pleiotropic functions of TIMP1 and TIMP2 [14].

As major findings of our study we highlighted that biomarkers found in seminal fluids represent the endpoint of a complex liquefaction proteolytic cascade that involves matrix metalloproteinases MMP3, MMP7 and MMP14 particularly when associated with down-regulation of TIMP1. Downregulation of TIMP1 and TIMP2 has been found by in situ hybridization in the stroma of PCa with higher Gleason scores (GS 8–10) compared to tissue of low Gleason scores [15], supporting our immunohistochemical findings. MMPs are generally more active in advanced stages, caused either by upregulation of MMPs and/or downregulation of their specific TIMPs and MMP activity has been linked to metastasis [16]. MMPs can interfere with growth signals, inhibit apoptosis, and induce angiogenesis and lymphangiogenesis all promoting tumor progression and metastasis. However, MMPs may also possess nonproteolytic functions, like triggering cell migration by chemotaxis (MMP14/TIMP2-MMP2 complex) or interfering with the complement proteinase cascade by interaction with C1q [17]. In line with existing literature, we found significant downregulation of TIMP1 in PCa-gp3, while downregulation of TIMP2 did not reach significant levels. TIMP1- and TIMP2-levels were also lower in PCa-gp4 compared to BPH (Figure 4). In most cases, fluorescence intensity was significantly higher in epithelial cells (EC) than in interstitial smooth muscle cells (SMC), supporting the notion, that malignant transformation of epithelial cells is responsible for the altered protease and inhibitor levels. Further studies, using tumor or tumor stem cell specific markers are needed to analyze the subpopulations of epithelial cells and their contribution to the protease pattern [18].

Except for TIMP1 (Figure 3B and Figure 4H) gene expression and protein expression was not correlated. This is in line with the finding that the concordance between transcript levels and protein expression is only 48–64% in prostate specific proteome [19]. We used immunofluorescence techniques to study cellular protein expression levels. Therefore, variations in the protein detection might be due to weak antibody staining in the case of MMPs complex formation with TIMP1 and TIMP2 [20] or due to epitope masking [21]. 

Our findings that MMP3/TIMP1 and MMP7/TIMP1 ratios were significantly lower in BPH compared to nP and PCa (Figure 5A,B) is in line with the significant higher transcript expression levels in BPH (Figure 1B) and the higher protein expression in confocal analysis (Figure 4H,I). In consequence, activity of those two MMPs would be lower in BPH tissue, resulting in less cleavage activity in seminal plasma as well.

Although western blotting analysis of seminal plasma consistently detected KLK3, ACPP and MMP7 (Figure 2, lanes 3,4), the detection of CTSG, CMA1, MMP3, MMP14, TIMP1 and TIMP2 failed in seminal plasma even though they were detectable in prostate tissue samples. These results could reflect the complexation of MMPs with TIMPs that may mask the antibody recognition site as discussed above. Concerning CTSG and CMA1, it is well known that seminal plasma contains several specific CTSG inhibitors [22,23] and several classes of proteinases (e.g., Metalloproteinases, serine and cysteine proteinases) may be able to activate and degrade CTSG and CMA1 [24,25].

In case of MMP14 discrepancies between gene expression (significantly up regulated in BPH and PCa compared to nP) and protein tissue levels (significantly down regulated in PCa, compared to nP and BPH) cannot be explained by complexation with TIMP2. Our findings indicate that cells of the prostate interstitium (included in “total IF” columns) have increased protein expression and may account for levelling the decrease in protein expression in epithelial cells (Figure 4). Additionally, it should be taken into account that the protein content within cells considerably depends on secretion rates and secretory status. Thus, especially high MMP14 secretion rates from epithelial cells in PCa might account for the low levels of MMP14-IF measured in PCa tissues (Figure 4G).

In addition, we investigated seminal vesicles as a source of proteases and inhibitors. We found low-level expression of most proteases and, interestingly, we also detected TIMP1 and low levels of TIMP2 in seminal vesicles (Figure 3K,L).

So far, TIMP1 has been detected only in bovine tissue [26]. To our best knowledge, this study is the first evidence for TIMP expression in human seminal vesicles. Of noteworthy, TIMPs play an independent role beyond MMP inhibition, e.g., inhibition of tumor growth, invasion and metastasis, growth factor-like activity, inhibition of angiogenesis and suppression of programmed cell death [27,28]. TIMPs have also been detected in seminal plasma and peculiar secretion by human seminal vesicles has been suggested [29,30].

Our findings suggest that the proteinase cocktail provided for semen liquefaction varies considerably between nP (60.71 ± 6.80 years; mean ± SD), BPH (70.30 ± 6.45 years) and PCa (65.17 ± 6.83 years). Significant alterations in TIMP1 expression strongly suggests that altered proteinase activities could lead to formation of seminal protein fragments and could be a candidate for prostate cancer biomarkers. Although we did not directly measure MMP activity, the observed increases in MMP/TIMP-ratios might indicate disruption/degradation of physiological extracellular matrix associated to tumor growth. Interestingly, MMP3/TIMP1 and MMP7/TIMP1 ratios were not different in nP and PCa, while they were significantly lower in BPH (Figure 5). As the prevalence of BPH constantly rises with age affecting 50–70% in men in the 5th and 6th decade, respectively [31], it should be differentiated from PCa which occurs in the same age group. This view is supported by our previous study demonstrating the need of a two-step procedure using two different biomarker panels to reach accurate separation of groups: (1) 21PP for separation of PCa + BPH from CP + HC and (2) 5PP for final detection of PCa (PCa vs. BPH) [8].

At the moment the effect of alterations in single proteases cannot be clearly linked to the appearance of a specific cleavage product in seminal plasma due to unidentified co-players (e.g., endopeptidases) in the complex liquefaction cascade. However, elevated serum MMP7 levels were reported to be significantly associated with metastatic and advanced PCa and was therefore considered to be a biomarker candidate to detect metastatic PCa [32]. In addition, TIMP1 was found downregulated in adenocarcinoma compared to BPH [33] and loss of TIMP1 correlated with biochemical recurrence in patients with localized PCa [34]. These results are in line with our findings and support the idea of regulation of MMP activity by TIMPs as crucial event in PCa growth and progression.

**Figure 7:**
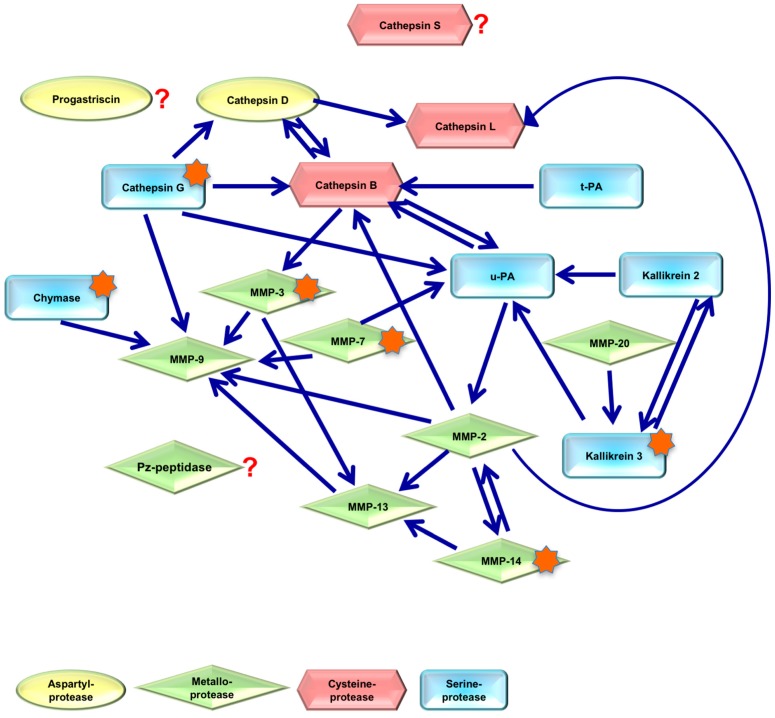
Proteolytic network in human seminal plasma. The role of some enzymes in liquefaction cascade is still obscure (?).

The complex network of proteinases found in the seminal plasma during prostate diseases (Figure 7) seems to generate many small proteolytic protein fragments, which represent the prostate cancer microenvironment and may be useful for both understanding prostate biology and as potent novel PCa biomarkers.

Semen liquefaction is the result of a complex proteolytic cascade involving different and multidirectional protease-protease interactions. Although the interactions involve proteinases from several families, at least three proteinases have been identified in seminal fluid with unknown direct/indirect interactions/functions with other protease classes (adaptation from reference data [13] and web databases: http://merops.sanger.ac.uk/, http://pmap.burnham.org/proteases; asterisks = proteinases with significant changes in MS/MS [8] and qPCR analyses (CMA1, MMP3, MMP7, MMP14, TIMP1, TIMP2; this study).

The major limitation of the present retrospective study was the restricted sample size. However, the aim of this study was to explore the role of MMP/TIMP network in prostate and seminal vesicles in the production of prostate cancer specific peptide patterns found in the seminal plasma. Future extended studies, including metastatic castration-resistant prostate cancer (CRPC) patients, are needed to validate this concept and to encourage the use of urine diagnostic markers as a tool for prostate cancer diagnosis and prognosis.

## 4. Material and Methods

### 4.1. Ethics Statement

The study was approved by the Ethics Committee of the University of Leipzig (Reg.No. 084-2009-20042009; 305-12-24092012) and was conducted according to the principles expressed in the Declaration of Helsinki. Written informed consent was obtained from all patients.

### 4.2. Quantitative Real-Time Polymerase Chain Reaction (qPCR)

We included samples from radical prostatectomies of patients with PCa (*n* = 12), transurethral resection of BPH (*n* = 10) and normal prostate tissue (nP; *n* = 7) after instantaneous section evaluation by our pathologist (LCH; Table 2).

Total RNA was extracted from deep frozen tissue specimens using peqGOLD TriFast extraction kit (peQLab, Erlangen, Germany) according to the manufacturers protocol and transcribed into cDNA using the Maxima First Strand cDNA Synthesis Kit (Fermentas, St. Leon-Rot, Germany). Quantitative PCR was performed with the real-time PCR-System realplex2 Mastercycler (Eppendorf, Hamburg, Germany) using the SYBR-Green quantitative PCR Mastermix (Fermentas) and custom primers (MWG-Biotech, Ebersberg, Germany; Table 3). Human 36B4 (acidic ribosomal phosphoprotein P0) served as internal standard for normalization using the 2^−∆∆*C*t^ method for relative quantification [35].

### 4.3. Protein Analysis by Indirect Immunofluorescence

We performed semi-quantitative analyses of the immunofluorescence (IF) and distribution of target proteins in tissue samples of normal prostate (*n* = 6), BPH (*n* = 6), seminal vesicles (*n* = 6) and PCa (*n* = 10; Table 4) using confocal laser scanning microscopy (CLSM).

Seven micron thick paraffin sections of prostate biopsies and material from radical prostatectomies were deparaffinized, rehydrated and processed for antigen retrieval in 10 mM citrate buffer (pH 6.0, 100 °C) in a steamer (Braun, Kronberg, Germany). Slices were then washed in phosphate-buffered saline (pH 7.4) and transferred to Tris-buffered saline (50 mM TBS, pH 7.4). Following treatment with TBS (0.1% Triton X-100) for 10 min and blocking unspecific binding (TBS, 0.1% Triton X-100, 1% bovine serum albumin, 3% fat-free milk powder) for 15 min at room temperature, slices were incubated overnight in a cocktail of anti-alpha smooth muscle cell actin monoclonal mouse IgG2a antibody (1:2000) and target antibody in blocking buffer at 4 °C (Table 5). 

For visualization, the sections were incubated with Alexa Fluor^®^ 488 goat-anti-mouse IgG2a and Alexa Fluor^®^ 555 goat anti-rabbit antibodies (Invitrogen, Karlsruhe, Germany) at a dilution of 1:500 for 1 h at room temperature. For semi-quantitative analysis scans were acquired at a LSM5 Pascal (Carl Zeiss, Jena, Germany) using a Plan-Neofluar 20x/0.5 Objective (Carl Zeiss, Jena, Germany) at 488 and 543 nm excitation wavelengths. Pinholes were adjusted to give an optical slice of <5.0 µm. Hematoxylin/Eosin stained sections were evaluated by the pathologist and regions of defined histological grading were defined. Five images were taken randomly in corresponding immunofluorescence stained sections and analysed in ImageJ 1.49t (Rasband WS. ImageJ. U S National Institutes of Health, Bethesda, Maryland, USA, available online: http://rsb.info.nih.gov/ij/, 1997–2006) using self-written analysis tools. Data were transferred to Apache OpenOffice™ 3 (Apache Software Foundation, available online: https://www.apache.org) for calculations. Target mean fluorescence intensities were normalized to staining control (omission of primary antibody) fluorescence intensities.

### 4.4. SDS Page and Western Blotting

We used six samples of normal prostate tissue that was excised following radical resection of the prostate and examined by our pathologist (LCH) to ensure tumor-free samples. Seminal plasma samples were collected and stored in liquid nitrogen at −196 °C until use. For SDS-PAGE (sodium dodecyl sulphate polyacrylamide gel electrophoresis), prostate tissue specimens were thawed on ice and later extracted using 500 µL/100 mg 50 mmol/L Tris-HCL supplemented with 1% sodium dodecyl sulphate (SDS), 5 µL protease inhibitor cocktail (Sigma-Aldrich, Steinheim, Germany) and 5 µL phenylmethylsulfonylfluoride. The tissue was homogenized on ice using an Ultra Turrax T10 (Ika GmbH & Co., Staufen, Germany), further extracted for 1 h at 4°C and thereafter centrifuged at 500× *g* for 2 min at 4 °C. Protein concentration of the supernatant was determined by Pierce™ BCA Protein Assay Kit (Thermo Fischer Scientific, Rockford, IL, USA) using a NanoDrop 1000 spectrophotometer (PEQLAB Biotechnologie GmbH, Erlangen, Germany). Seminal plasma probes were thawed on ice and protein concentration was determined as indicated above. 30 or 50 µg protein was used per lane on NuPage Bis-Tris 4–12% gradient mini gels (Life Technologies, Darmstadt, Germany).

Western blotting was performed onto PVDF membranes according to supplier’s protocol in a Bio-Rad Mini Trans-Blot^®^ system (Bio-Rad Laboratories GmbH, München, Germany). Membranes were blocked for 1 h with 2% BSA (Carl Roth GmbH & Co. KG, Karlsruhe, Germany) followed by incubation with primary antibody incubation at 4 °C (Table 5). Anti-beta actin and anti-GAPDH antibodies were used as loading controls. For detection, we used goat-anti mouse IRDye 680 or goat-anti rabbit IRDye 680 secondary antibodies diluted at 1:8000 (LI-COR Biosciences GmbH, Bad Homburg, Germany). Blots were scanned using an Odyssey Infrared Imaging System (LI-COR).

### 4.5. Statistical Analysis

Statistics and graphing were done with Prism 5 (GraphPad Software Inc., La Jolla, CA, USA). Parameter free tests were used as indicated in the figure legends. All numbers are given as mean ± SEM (standard error of the mean), unless indicated otherwise.

## 5. Conclusions

We conclude that tissue levels of MMP3 and MMP7 (finely regulated by their inhibitors TIMP1 and TIMP2) are altered in PCa compared to benign prostatic hyperplasia, which is of special relevance in elderly men. These findings link tissue alterations to altered liquefaction cascade, indicating disease-related alterations in prostate tissue and hence in liquefaction cascade. For the first time, we present evidence for a contribution of seminal vesicles to the fine-tuning of the liquefaction protease network. Our findings thus support the concept of seminal plasma biomarkers as non-invasive tool for PCa detection and risk stratification in men aged 60–70 years.

## Figures and Tables

**Table 1:** Semenogelin derived seminal peptides and their putative proteolytic cleavage sites.

Peptide ID	PCa Biomarker	Peptide Sequence	Peptide Identification	Cleavage Site	Putative Protease
15331	Yes	YVLQTEELVVNKQQRETKNSHQ	SEMG2 (194-215)	SQSS//YVLQ	MMP3, MMP7, MMP13, MMP14, MMP20
1677	No	VLQTEELVA	SEMG1 (195-203)	QSSY//VLQT	KLK3
6925	No	VLQTEELVANKQQ	SEMG1 (195-207)	QSSY//VLQT	KLK3
7186	No	VLQTEELVVNKQQ	SEMG2 (195-207)	QSSY//VLQT	KLK3
10289	No	VLQTEELVANKQQRET	SEMG1 (195-210)	QSSY//VLQT	KLK3
11260	No	VLQTEELVVNKQQRETK	SEMG2 (195-211)	QSSY//VLQT	KLK3
11899	Yes	TEELVANKQQRETKNSHQ	SEMG1 (198-215)	YVLQ//TEEL	KLK3
12083	Yes	TEELVVNKQQRETKNSHQ	SEMG2 (198-215)	YVLQ//TEEL	KLK3
18990	Yes	SQTEEKAQGKSQKQITIPSQEQEHSQKAN	SEMG1 (316-344)	SSIY//TEEL	KLK3, CMA1, CTSG

Peptide sequences including LC-MS/MS data have been recently published by our group [8]. ID: polypeptide identifier annotated by the structured query language (SQL) database (ID); known cleavage sites are indicated for semenogelin-1 (P04279, Swiss-Prot Acc.No.) and semenogelin-2 (Q02383) with their putative proteases, as supported by literature research of web databases: http://merops.sanger.ac.uk/, http://pmap.burnham.org/proteases and http://www.proteolysis.org/proteases.

**Table 2:** Characteristics of patients used for qPCR analysis.

Group	*n*	Age (Mean ± SD) (95% CI)	PSA [ng/mL] (95% CI)	Gleason Score	Histology
nP	7	60.71 ± 6.80 (54.43–67.00)	n.a.	n.a.	normal prostate
BPH	10	70.30 ± 6.447 (65.69–74.91)	n.a.	n.a.	BPH
PCa	12	65.17 ± 6.834 (60.82–69.51)	6.097 ± 1.868 (4.910–7.284)	≤6 (*n* = 6) 7 (*n* = 2) >7 (*n* = 4)	pT2a (*n* = 2) pT2c (*n* = 7) pT3a (*n* = 3)

Age was not significantly different between groups (*p* = 0.0556; *Kruskal–Wallis* test, *Dunn’s* Multiple Comparison Test). PSA values were not available for nP and BPH patients (n.a.).

**Table 3:** Primer pairs used for qPCR analysis of the liquefaction cascade. KLK3 (PSA): kallikrein related peptidase 3; MMP: matrix metalloproteinase; CMA1: chymase 1; CTSG: cathepsin G; ACPP: prostatic acid phosphatase; h36B4: ribosomal protein P0 (housekeeping gene).

Gene	Acc. No.	Sequence (F) forward, (R) reverse	Binding to Exon
*ACPP* (both isoforms)	NM_001099.4	(F) 5′-cga agt ccc att gac acc tt-3′	2
		(R) 5′-atc aaa gtc cgg tca acg tc-3′	4
*ACPP-V1* (transcript variant 1)	NM_001099.4	(F) 5′-tgt gag tgg cct aca gat gg-3′	9
		(R) 5′-tgt act gtc ttc agt acc ttg a-3′	10
*ACPP-V2* (transcript variant 2)	NM_001134194.1	(F) 5′-gga ctc ctt cct ccc tat gc-3′	9
		(R) 5′-agg caa cag caa aga tga cc-3′	11
*PSA/KLK3*	NM_001030047.1	(F) 5′-cat gct gtg aag gtc atg ga-3′	3
		(R) 5′-agc aca cag cat gaa ctt gg-3′	4
*MMP3*	NM_002422.1	(F) 5′-gca gtt tgc tca gcc tat cc-3′	1
		(R) 5′-gag tgt cgg agt cca gct tc-3′	2
*MMP7*	NM_002423.3	(F) 5′-gag tgc cag atg ttg cag aa-3′	2
		(R) 5′-gcc aat cat gat gtc agc ag-3′	3
*MMP13*	NM_002427.3	(F) 5′-ttg agc tgg act cat tgt cg-3′	1
		(R) 5′-gga gcc tct cag tca tgg ag-3′	2
*MMP14*	NM_004995.2	(F) 5′- caa gca ttg ggt gtt tga tg-3′	8
		(R) 5′-tcc ctt ccc aga ctt tga tg-3′	9
*MMP20*	NM_004771.3	(F) 5′-ctc atc ctt tga cgc tgt ga-3′	6
		(R) 5′-ctt cgt aag ctg cat cca ca-3′	7
*CMA1*	NM_001836.2	(F) 5′-tgc aag agg tga agc tga ga-3′	4
		(R) 5′-gag att cgg gtg aag aca gc-3′	5
*CTSG*	NM_001911.2	(F) 5′-ata atc agc gga cca tcc ag-3′	3
		(R) 5′-tgc cta tcc ctc tgc act ct-3′	4
*h36B4*	NM_002775.1	(F) 5′-ccg act cct ccg act ctt c-3′	6
		(R) 5′-aac atg ctc aac atc tcc cc-3′	8

**Table 4:** Characteristics of patients used for immunofluorescence analysis.

Group	*n*	Age (95% CI)	PSA [ng/mL] (95%CI)	Gleason Score	Histology
nP	6	62.17 ± 4.79 (57.14–67.20)	n.a.	n.a.	normal prostate
BPH	6	67.50 ± 5.93 (61.28–73.72)	n.a.	n.a.	BPH
PCa	10	61.50 ± 9.25 (54.88–68.12)	19.44 ± 12.62 (*n* = 8)	6 (*n* = 2) 7 (*n* = 8)	pT1c (*n* = 7) pT2c (*n* = 3)
SV	6	61.17 ± 8.68 (52.06–70.28)	11.41 ± 4.87 (*n* = 5)	6 (*n* = 2) 7 (*n* = 4)	normal, not infiltrated

Age was not significantly different between groups (*p* = 0.3997; *Kruskal–Wallis* test, *Dunn’s* Multiple Comparison Test). PSA values were not available for nP and BPH patients (n.a.).

**Table 5:** Antibodies used in indirect immunofluorescence (IF) and Western blotting (WB). Sources: ^1^ Acris Antibodies GmbH, Herford, Germany; ^2^ antibodies online, Aachen, Germany; ^3^ Santa Cruz Biotechnology Inc., Santa Cruz, CA, USA; ^4^ Proteintech Group Inc., Manchester, UK; ^5^ Histo-line Laboratories, Milan, Italy; ^6^ Abcam Inc., Cambridge, MA, USA; ^7^ QED Bioscience Inc., San Diego, CA, USA; ^8^ Novus Biologicals Europe, Abingdon, UK; ^9^ Sigma-Aldrich Chemie GmbH, Steinheim, Germany; ^10^ LI-COR Biosciences GmbH, Bad Homburg, Germany; Rb (rabbit); Ms (mouse); polyclonal (poly); monoclonal (mono).

Antigen/Primary Antibodies	Source	Type	Order-No.	Dilution
KLK3	1	Rb, poly	AP15748PU-S	1:200 (IF), 1:500 (WB)
CMA1	2	Rb, poly	ABIN679853	1:200 (IF), 1:400 (WB)
CTSG	2	Rb, poly	ABIN731843	1:200 (IF), 1:500 (WB)
MMP3	2	Rb, poly	ABIN668301	1:200 (IF)
MMP3	3	Ms, mono	sc-21732	1:500 (WB)
MMP7	2	Rb, poly	ABIN668451	1:200 (IF)
MMP7	4	Rb, poly	10374-2-AP	1:500 (WB)
MMP14	5	Rb, poly	29025	1:200 (IF)
MMP14	6	Rb, poly	ab3644	1:500 (WB)
ACPP-V1	2	Rb, poly	ABIN966903	1:80 (IF), 1:500 (WB)
TIMP1	7	Rb, poly	29022	1:200 (IF), 1:1000 (WB)
TIMP2	2	Rb, poly	ABIN373976	1:200
β Actin	8	Ms, mono	NB600-501	1:4000 (WB)
GAPDH	3	Ms, mono	sc-47724	1:1000 (WB)
alpha-smooth muscle actin	9	Ms, mono	A2547	1:2000
goat-anti Ms IRDye^®^ 680RD	10	Goat IgG	926-68070	1:8000 (WB)
goat-anti Rb IRDye^®^ 680RD	10	Goat IgG	926-68071	1:8000 (WB)
